# Genome-wide analysis of signatures of selection in populations of African honey bees (*Apis mellifera*) using new web-based tools

**DOI:** 10.1186/s12864-015-1712-0

**Published:** 2015-07-10

**Authors:** Zachary L. Fuller, Elina L. Niño, Harland M. Patch, Oscar C. Bedoya-Reina, Tracey Baumgarten, Elliud Muli, Fiona Mumoki, Aakrosh Ratan, John McGraw, Maryann Frazier, Daniel Masiga, Stephen Schuster, Christina M. Grozinger, Webb Miller

**Affiliations:** Department of Biology, Pennsylvania State University, University Park, PA USA; Department of Entomology, Center for Pollinator Research, Pennsylvania State University, University Park, PA USA; Center for Comparative Genomics and Bioinformatics, Pennsylvania State University, University Park, PA USA; Department of Biological Sciences, South Eastern Kenya University (SEKU), P.O. Box 170-90200, Kitui, Kenya; The International Center of Insect Physiology and Ecology (icipe), PO Box 30772-00100, Nairobi, Kenya; Department of Biochemistry and Molecular Biology, Pennsylvania State University, University Park, PA USA

**Keywords:** Apis mellifera, Galaxy, Genome sequencing, Adaptive evolution

## Abstract

**Background:**

With the development of inexpensive, high-throughput sequencing technologies, it has become feasible to examine questions related to population genetics and molecular evolution of non-model species in their ecological contexts on a genome-wide scale. Here, we employed a newly developed suite of integrated, web-based programs to examine population dynamics and signatures of selection across the genome using several well-established tests, including *F*_ST_, pN/pS, and McDonald-Kreitman. We applied these techniques to study populations of honey bees (*Apis mellifera*) in East Africa. In Kenya, there are several described *A. mellifera* subspecies, which are thought to be localized to distinct ecological regions.

**Results:**

We performed whole genome sequencing of 11 worker honey bees from apiaries distributed throughout Kenya and identified 3.6 million putative single-nucleotide polymorphisms. The dense coverage allowed us to apply several computational procedures to study population structure and the evolutionary relationships among the populations, and to detect signs of adaptive evolution across the genome. While there is considerable gene flow among the sampled populations, there are clear distinctions between populations from the northern desert region and those from the temperate, savannah region. We identified several genes showing population genetic patterns consistent with positive selection within African bee populations, and between these populations and European *A. mellifera* or Asian *Apis florea*.

**Conclusions:**

These results lay the groundwork for future studies of adaptive ecological evolution in honey bees, and demonstrate the use of new, freely available web-based tools and workflows (http://usegalaxy.org/r/kenyanbee) that can be applied to any model system with genomic information.

**Electronic supplementary material:**

The online version of this article (doi:10.1186/s12864-015-1712-0) contains supplementary material, which is available to authorized users.

## Background

Understanding the molecular mechanisms regulating individual variation and how selection operates on these mechanisms to drive adaptive evolutionary change are fundamental goals in evolutionary ecological genetics [1, 2]. However, studies of the molecular mechanisms mediating intra- and interpopulation differences in non-model organisms in their ecological habitats have traditionally been limited by the relatively low number of genetic markers available and the high cost of full genomic sequencing. The introduction of inexpensive, high-throughput sequencing technologies presents the unparalleled opportunity to rapidly analyze the structure of populations in ecologically relevant systems and begin to examine the evolutionary forces underpinning adaptive variation within and among populations and species [3, 4].

There are a large number of well-established computational procedures and statistical techniques to detect signatures of selection along the genome at different evolutionary time periods (see Sabeti, et al. 2006 [5]). However, the majority of these methods are computationally intensive for genome-wide analyses and difficult to execute for researchers not accustomed to working with population genomic data and the associated statistical techniques. Recently, we developed a user-friendly, open-access, web-based platform on the Galaxy web-server to investigate genetic variation and population structure [6]. In this study, we expand upon this existing platform through the development of additional tools and reproducible workflows to specifically scan the genome for signatures of selection and adaptive evolution. This interface allows scientists to easily and efficiently examine the evolutionary processes acting on population-genomic data sets and readily share their results with the scientific community, ensuring transparency and reproducibility.

Here, we demonstrate an application of this platform in the study of evolutionary relationships among populations of *Apis mellifera* honey bees in East Africa, and search for signs of adaptive evolution across the genome. Honey bees are a fascinating model system in which to study the molecular mechanisms underpinning adaptive evolution. *Apis mellifera* honey bees are one of the most broadly dispersed animal species in the world, occupying every continent except Antarctica. *A. mellifera* originally evolved in tropical Africa, and subsequently migrated to and became established in temperate Europe and western Asia on two occasions, forming two distinct genetic lineages from their African ancestors [7–9]. Within Kenya alone there is substantial genetic diversity, with previous studies identifying five distinct subspecies in different ecological niches: *A.m. scutellata* in the central savannah, *A.m. monticola* in the mountains, *A.m. litorea* on the coast, *A.m. yemenitica* and *A.m. simensis* in the northern desert [7, 9–11]. However, previous studies using a limited number of mitochondrial and nuclear markers have indicated substantial gene flow among these putative subspecies, and thus there may not be clear distinctions among these populations [12, 13].

Adaptation to different environmental conditions in honey bees involves both behavioral and physiological changes. Honey bees establish large colonies with tens of thousands of individuals which are active year-round [14]. In tropical regions, colonies must deal with both wet and dry seasons, while in temperate regions, colonies must survive extreme cold conditions. Winter in temperate regions and either (depending on the conditions) wet or dry seasons in tropical regions are typically associated with reduced floral resources and reduced nutrient intake, which results in reduced production of new bees (brood). In temperate regions, this nutrient-deprived, broodless condition has been shown to trigger the production of “overwintering bees” [15], which exhibit reduced activity, altered hormone profiles, higher nutrient stores, and longer lifespans [16–18]. The bees will also form a thermoregulating cluster when temperatures drop below 18 °C [14]. The phenotype of bees experiencing wet/dry conditions in the tropics has not been characterized, but rendering temperate bees broodless in the summer months can result in a similar “overwintering” phenotype of altered hormone and nutrient levels and increased lifespans [17, 19–21], and it has been hypothesized that broodless conditions resulting from a lack of resources can trigger similar phenotypes under different environmental conditions [15, 22]. Furthermore, in addition to these physiological parameters, there are also clear behavioral adaptations to seasonal changes: colonies of tropical *A. mellifera* subspecies readily migrate over vast distances to find environments with more plentiful resources [23].

With the sequencing of the honey bee genome [24], and the development of high throughput genomic sequencing technologies, it is now possible to readily examine genome-wide signatures of selection in honey bees. Previous studies have examined the genetic structure of populations of bees in Africa, Europe and North America allowing for inferences of the phylogenetic history of worldwide honey bee populations and identifying signatures of positive selection in protein coding regions of genes between African/Africanized populations and European populations [8, 25, 26]. Other studies demonstrated that genes associated with differences in queen and worker behavior have significantly higher numbers of SNPs than uncorrelated genes [27], and identified signatures of postive selection in genes associated with worker behavioral traits [28].

In this study, we sequenced the full genomes of 11 individual worker bees sampled from different ecological regions throughout Kenya. We expanded and used our newly developed suite of web-based tools to examine signatures of selection within this sample group using six well established tests; each test is available as a tool or reproducible workflow on the Galaxy website, http://usegalaxy.org/r/kenyanbee. As with the experimental design of any population genetics analysis, large sample sizes are always desired and lead to the greatest accuracy in the estimation of population genetics parameters [29]. Despite the decreasing price of high throughput technologies, the costs of whole genome sequencing, sample collection and international field work remain as limitations to population based genomics studies. We acknowledge the potential limitations our relatively small size of 11 individuals yield, yet demonstrate the powerful application of publicly accessible, user-friendy tools developed on the Galaxy platform to begin to identify the genes involved in environmental adaptation in this species. Additionally, our work provides the foundation and computational framework for future population genomics studies in Kenyan honey bees, as well as other ecologically relevant non-model organisms.

## Methods

### Sample collection

We collected honey bees from 11 apiaries across Kenya (these bees were sampled as part of a larger study surveying bee health in 24 apiaries across Kenya, described in [12]. Detailed information about the location and levels of parasites/pathogens in each apiary is supplied in Additional file 1: Table S1. All samples were collected at maintained apiaries and consent was given by the collaborating authority. In all cases the owner (in the case of private land) or relevant authority (in the case of public land, gave permission for collections). Honey bees were maintained according to standard beekeeping practices. One representative bee from one colony in each apiary was sampled; collections of bees were carried out between June-September 2010. A forager returning to the hive entrance with pollen was collected in 95 % ethanol. Bees were collected into individual 2 ml cryogenic vials (VWR, Radnor, PA). Samples were collected on ice, stored at -20 °C during field collections, and then shipped to The Pennsylvania State University (University Park, PA) within a month, where they were stored at 4 °C. Sample collection procedures were in accordance with standard practices for ethical handling of invertebrate samples [30]. Because *A. mellifera* is not a regulated invertebrate, no ethical use or institutional review board approval was required.

### W*hole genome sequencing and SNP identification*

Heads of individual foragers were dissected and homogenized with a Fastprep instrument (Thermo Fisher, Waltham, MA) for three cycles at maximum time and speed. DNA was extracted using the Puregene Core Kit (Qiagen, Valencia, CA) according to manufacturer’s instructions.

Prior to library preparation, the quality of the gDNA samples was assessed by running the samples on a Bioanalyzer DNA 12000 Chip (Agilent, Santa Clara, CA). Sample quantitation was performed using Invitrogen’s Picogreen assay. Next-generation sequencing library preparation was performed on the Biomek FXp (Beckman, Brea, CA) using the SPRIworks HT Reagent Kit (Beckman) and Illumina’s TruSeq LT DNA adatpers (Illumina, San Diego, CA). For each sample, 1ug of gDNA was used for library preparation. The samples were sheared on a Covaris S2 to ~300 bp, following the manufacturer’s recommendation (Covaris, Woburn, MA). Size selection was performed on the Biomek FXp using the SPRIworks HT Reagent Kit. Each library was uniquely tagged with one of Illumina’s TruSeq LT DNA barcodes to allow library pooling for sequencing. Library quantitation was performed using Invitrogen’s Picogreen assay and the average library size was determined by running the libraries on a Bioanalyzer DNA 1000 chip (Agilent). Library concentration was validated by qPCR on a StepOne Plus real-time thermocycler (Applied Biosystems, Grand Island NY), using qPCR primers, standards and reagents from Kapa Biosystems (Wilmington, MA). Library quality was assessed by running the samples on an Illumina MiSeq sequencer and high throughput sequencing was carried out on an Illumina HiSeq 2000 sequencer at a read-length of 101 bp paired-end.

Paired-end sequences of length 101x100 bp were aligned to the *Apis mellifera* reference genome sequence (Amel_4.5) using BWA [31] version 0.5.9. The default parameters were used, with the exception of the “-q 15” option, which was applied to allow soft trimming of the low-quality 3’ ends of reads prior to alignment. On average, we aligned 5.06 Gb of sequence data per individual (SD 1.68 Gb), corresponding to an average of ∼ 20-fold coverage of the 234-Mb honey bee reference genome sequence. We used the MarkDuplicates utility in the Picard toolset (http://picard.sourceforge.net) to flag potential PCR duplicate reads that could otherwise affect the quality of the variant calls. Of each set of potential duplicate read pairs, only the pair with the highest sum of base quality scores for bases with quality ≥15 was used in the subsequent steps. Considering data from all individuals simultaneously, we used SAMtools [32] version 0.1.18 to identify the locations of variants, using the option “-C 50” to reduce the mapping quality of the reads with multiple mismatches. These locations in the nuclear genome were filtered to maintain variants for which the total coverage in the samples was between 4 and 500 reads (to limit the erroneous calling of variant positions in repetitive or duplicated regions), and the RMS (root mean square) mapping quality was greater than or equal to 10. As a result, we identified 8,363,799 locations (6,735,513 SNPs, and 1,628,286 small indels) in the nuclear genome, where more than one allele was observed among the 11 samples and the reference sequence. Once the variant locations were identified, we then used SAMtools (using the mpileup command) to estimate genotypes at all SNPs for each individual, regardless of sequence coverage for that SNP and individual. The final dataset of 3,643,069 putative SNPs was constructed after filtering for a SAMtools-computed quality score of at least 100. All sequences are deposited on the short read archive (Accession Number: SRP037570) and as a BioProject (Accession Number: PRJNA237819) on NCBI.

### Overall F_ST_

Samples were collected from apiaries that had previously been found to contain bees with mitochondrial haplotypes corresponding to four previously described subspecies (*A. m. litorea, A. m. yemenitica, A. m. scutellata* and *A. m. monticola*) though in some cases, multiple subspecies were identified within an apiary (see Table 1, Additional file 1: Table S1 and [12])*.* To determine if there were significantly differentiated populations of honey bees within our sample group using the whole genome information, we generated 10,000 randomized groupings of individuals to be compared using the Reich-Patterson estimator of *F*_ST_ [33]. Two groupings were identified (see Results) which we termed “Desert” and “Savannah”. Both the data randomization and *F*_*ST*_ estimation were performed using the “Overall *F*_*ST*_” tool available in the “Genome Diversity” toolset on Galaxy (http://usegalaxy.org).

**Table 1:** Specimen location information

Site	Geographic region	Site Name	Coordinates	Elevation	Individual ID	Köppen-Geiger classification^a^	Parasites & Pathogens^b^
1S	savannah	Nairobi, Kasarani	−1°13.3631, 36°53.7867	1602 m	1.4.15	Cfb-Aw	VD
2S	savannah	Kitui, SEKU	−1°18.3005, 37°45.9075	1150 m	2.2.15	Aw	DWV
3S	savannah	Malewa	−0°31.5024, 36°24.1969	1973 m	4.2.15	Csb	VD, DWV
4S	savannah	Mt. Elgon, Chepkui	0°49.543, 34°42.1740	1869 m	21.3.15	Am	VD, BQCV, DWV
1C	coast	Gete Ruins	−3°18.3899, 40°1.0793	36 m	12.2.15	Aw	VD, BQCV, DWV, N. apis
2C	coast	Oceanside	−3°20.2514, 39°59.1495	15 m	13.4.15	Aw	VD, DWV, ABPV, N. apis
3C	coast	Tanzania Border	−4°31.7394, 39°9.2171	62 m	15.4.25	Aw	VD
1D	desert	Mandera Town 1	3°56.2050, 41°52.0900	212 m	16.1.5	BWh	No Pathogens
2D	desert	Mandera Town 2	3°56.1970, 41°52.0820	221 m	17.1.5	BWh	No Pathogens
3D	desert	Mandera West	3°53.3790, 40°16.0440	894 m	18.1.5	BSh	No Pathogens
1 M	mountain	Mt. Elgon, Moorland	0°57.2460, 34°36.2860	2956 m	22.2.5	Am	BQCV

^a^Am-Tropical monsoon climate, Aw- Tropical savannah climate, Bsh- Subtropical steppe climate, Bwh- Subtropical desert climate, Cfb- Marine coastal climate, Csb- Mediterranean climate

^b^ABPV- Acute bee paralysis virus, BQCV- Black queen cell virus, DWV- Deformed wing virus, N.apis- Nosema apis, VD-Varroa destructor

The site number, geographic region, site name, coordinates, elevation, and identification numbers for individual specimens used in this study with Köppen-Geiger classification (Peel et al. 2007) [106] and previous identified pathogens at each site (Muli, et al. 2014) [12]. The apiary, colony and individual identification (ID) numbers were assigned based on our initial survey scheme (Muli, et al. 2014) [12]

### Phylogenetic analysis

The complete nuclear and mitochondrial sequences from the 11 individuals were used for phylogenetic analysis. The nuclear genome phylogeny was generated using the filtered set of ~3.6 million SNPs with the “Phylogenetic Tree” tool located under the “Genome Diversity” section on Galaxy (http://usegalaxy.org). “Phylogenetic Tree” constructs a neighbor-joining tree using the QuickTree program [34].

Complete mitochondrial genome sequences were aligned with the ClustalW package in BioEdit 7.6 [35] using the mutiple alignment feature (BLOSUM62 matrix, gap open penalty: 15.0, gap extension penalty: 6.66). A neighbor-joining tree was constructed with MEGA5 [36] using the Maximum Composite Likelihood model (2000 bootstrap replicates), with uniform rates, and complete deletions. There were 12 sequences in all, including the outgroup individual, with 16,051 nucleotides in the final data set of which 456 were variable sites. The European subspecies *Apis mellifera ligustica* (Accession Number: L06178) is used as the outgroup to the African bees. The percentage of replicate trees in which the associated taxa clustered together in the bootstrap test are shown next to the branches [37].

### pN/pS

Using the honey bee reference genome (Amel v4.5; [38]) we identified the annotated protein-coding exons for each putative gene. For each protein coding region, we analyzed all codons, other than the stop codon or those that intersect a gap (run of the letter N) in the assembly. We defined the counts of effective nonsynoymous and synonymous sites using the approach of Nei [39]. We then constructed a table containing the number of synonymous and nonsynonymous polymorphsims for each gene. A table based on all putative SNPs and the associated workflow is available on the Galaxy server (http://usegalaxy.org/r/kenyanbee).

### Fixed differences from the (European) reference genome

The number of nonsynonymous and synonymous SNPs that are invariant among our samples and differ from the *Apis mellifera* reference (which was derived from a European honey bee subspecies), [24] can be computed for each reference gene with Galaxy commands and we provide a workflow at the Galaxy website, http://usegalaxy.org/r/kenyanbee, that computes all of the values reported herein as well as the related supplementary table.

### McDonald-Kreitman test

We extracted intervals of the *A. mellifera* reference genome that include coding exons plus 10 flanking bases on each side, which were then aligned to the *A. florea* reads using BWA and default parameters [31]. This allowed the McDonald-Kreitman test to be applied to all 15,314 annotated *A. mellifera* genes within our Kenyan samples. The number of synonymous subsitutions (D_S_), the number of nonsynonymous substitutions (D_N_), the number of synonymous polymorphisms (P_S_) and the number of nonsynonymous polymorphisms (P_N_) were counted using the Galaxy “filter” and “count” commands; the Galaxy table of bee genes that reports pN/pS (mentioned above) also contains columns with the MK ratio, D_S_, D_N_, P_S_ and P_N_. The associated tools and workflow are available at the Galaxy website, http://usegalaxy.org/r/kenyanbee.

### Runs of homozygosity

Allele frequencies were calculated using the “filter”, “count” and “text manipulation” tools available on the Galaxy browser. For each defined population, either Desert or Savannah, columns were appended to the data table that summed the genotypes for all individuals specified. Using the “compute” tool, a homozygosity score was given for each site by taking the square of the difference of the frequencies of the alternate and reference alleles. In this way, any site that is homozygous within a population will receive a score of 1.0. With the “Remarkable Intervals” tool on the Galaxy Browser, intervals of consecutive homozygous genotypes, or runs of homozygosity (ROH), were discovered by setting a cutoff of 0.9 (a cutoff of 1.0 would not allow for the “Remarkable Intervals” tool to extend intervals from a single site). We used 1000 randomizations to determine which intervals to report, so that for any interval reported, the probability is *p* < 0.001 for that interval’s score being equaled or exceeded by chance. These intervals were then intersected with and joined to known genes using the “intersect” and “join” commands on Galaxy. A workflow showing the steps and tools used is available on Galaxy (http://usegalaxy.org/r/kenyanbee).

### F_ST_

We first selected individuals for either the Desert or Savannah population using the “Specify Individuals” tool on Galaxy. Using the “Per SNP *F*_*ST*_*’s*” tool*,* we then calculated the Reich-Patterson estimator of *F*_*ST*_ at each SNP and used the “Remarkable Intervals” tool to locate regions containing runs of consecutive SNPs with high *F*_*ST*_ values. We used the highest scoring 10 % of *F*_*ST*_ values as our cutoff. At these intervals, the Desert and Savannah populations are more different from one another than can be explained by chance alone (*p* < 0.001), demonstrated by our randomization approach using 1,000 permuted replicates. These intervals were then intersected with and joined to known genes using the “intersect” and “join” commands on Galaxy. A workflow showing the steps and tools used is available on Galaxy (http://usegalaxy.org/r/kenyanbee).

### Tajima’s D

Tajima’s *D* statistic was calculated according to the original definition [40]. *D* was calculated in 5 kb, non-overlapping windows across the entire genome of all individuals in the Kenyan sample using the “Tajima’s D” tool on Galaxy. For each 5 kb window, we also estimated the normalized version of Fay and Wu’s *H* statistic using a custom Python script [41, 42]. To correct for multiple testing, we used the experiment-wide simulation approach of Nielsen et al. (2005) [43]. Significance cutoffs were obtained by repeatedly simulating data from a neutral coalescence model using a value of θ estimated from the data by taking the average number of segregating sites in a window. Coalescent simulations were performed using Hudson’s *ms* and Tajima’s *D* and normalized Fay and Wu’s *H* values were estimated in sampled windows using Zeng’s *dh* program [41, 44]. We then filtered the data for windows with Tajima’s *D* scores more negative than the simulated significance cutoff (-1.71). A workflow showing the steps and tools used is available on Galaxy (http://usegalaxy.org/r/kenyanbee).

### Genomic intervals under selection

The methods that search for genomic intervals where the SNPs reveal signs of positive selection all use the Galaxy “Remarkable Intervals” tool and a workflow showing the steps and tools used is available on Galaxy (http://usegalaxy.org/r/kenyanbee). The tool avoids problems associated with a fixed window size and in essence automatically determines the sizes of the intervals that it finds. Certain genomic positions (e.g., SNPs) are assigned a numerical value (e.g., *F*_ST_), and the user specifies a “cutoff value” or percentile that exceeds most of these numbers. The tool subtracts the cutoff value from each number, then finds “locally optimal” genomic intervals where the sum of the subtracted values cannot be increased by adjusting the interval’s end-points. Also, it uses a randomization strategy to determine “empirical” *p*-values for the intervals that it reports. Detailed descriptions of the method are reported elsewhere [45].

### *Gene Ontology* (*GO*) *analysis*

Where possible, GO analysis was performed in DAVID for genes displaying patterns consistent with selection [46, 47]. GO analysis was carried out on the genes with *Drosophila* orthologs since DAVID software does not support honey bee genome annotation. For the background list (see Additional file 1: Table S2), we used all annotated *A. mellifera* genes with an available FlyBase annotation (8443). Numbers of genes in the analyzed gene lists with FlyBase orthologs were 39 for pN/pS, 102 for McDonald-Kreitman, 506 for Tajima’s *D*, and 60 for *F*_*ST*_. If Bonferroni or Benjamini procedures are applied to correct for multiple testing, no functional categories are identified as significant.

### Verification of SNPs in candidate genes

To validate our high throughput sequencing, selected genes (*Api m 6, FMRFamide receptor, NADH dehydrogenase*) were resequenced in the 11 individuals and in two individuals derived from European subspecies (*Apis melifera ligustica* and *Apis mellifera carnica*, obtained from Glenn Apiaries, Fallbrook, CA). DNA was extracted as above, and individual genes were amplified using PCR (see Additional file 1: Table S3 for list of genes and primers). PCR was performed on a Mastercycler Pro (Eppendorf, Hauppauge, NY) using 25 ml reactions consisting of 2.5 units of platinum Taq DNA Polymerase, PCR buffer minus magnesium at a concentration of 1X, 0.2 mM dNTP mix, 1.25 mM MgCl_2_, 5 % DMSO, 0.2 mM primers and 10 ng of extracted DNA; reagents were purchased from Invitrogen (Carlsbad, CA). The PCR was carried out using a touchdown protocol with the thermal profile of 2 minute at 95 °C, followed by 10 cycles of 95 °C (30 s), 65 (30 s, decreasing by 1 °C each cycle), and 72 °C (120 s). The program continued with 30 cycles of 95 °C (30 s), 55 °C (30 s) and 72 °C (120 s) and a final extension at 72 °C for 8 minutes. No-template controls were performed with each PCR run. Products were visualized by electrophoresis on 1.0 % agarose gels and were purified with a QIAquick PCR Purification Kit (Qiagen, Valencia, CA) for sequencing. Sequencing was performed by the Genomics Core Facility at Pennsylvania State University. Sequences were aligned using ClustalW package in BioEdit 7.6.

## Results and discussion

### Evolutionary relationships among Kenyan honey bee populations

**Fig. 1:**
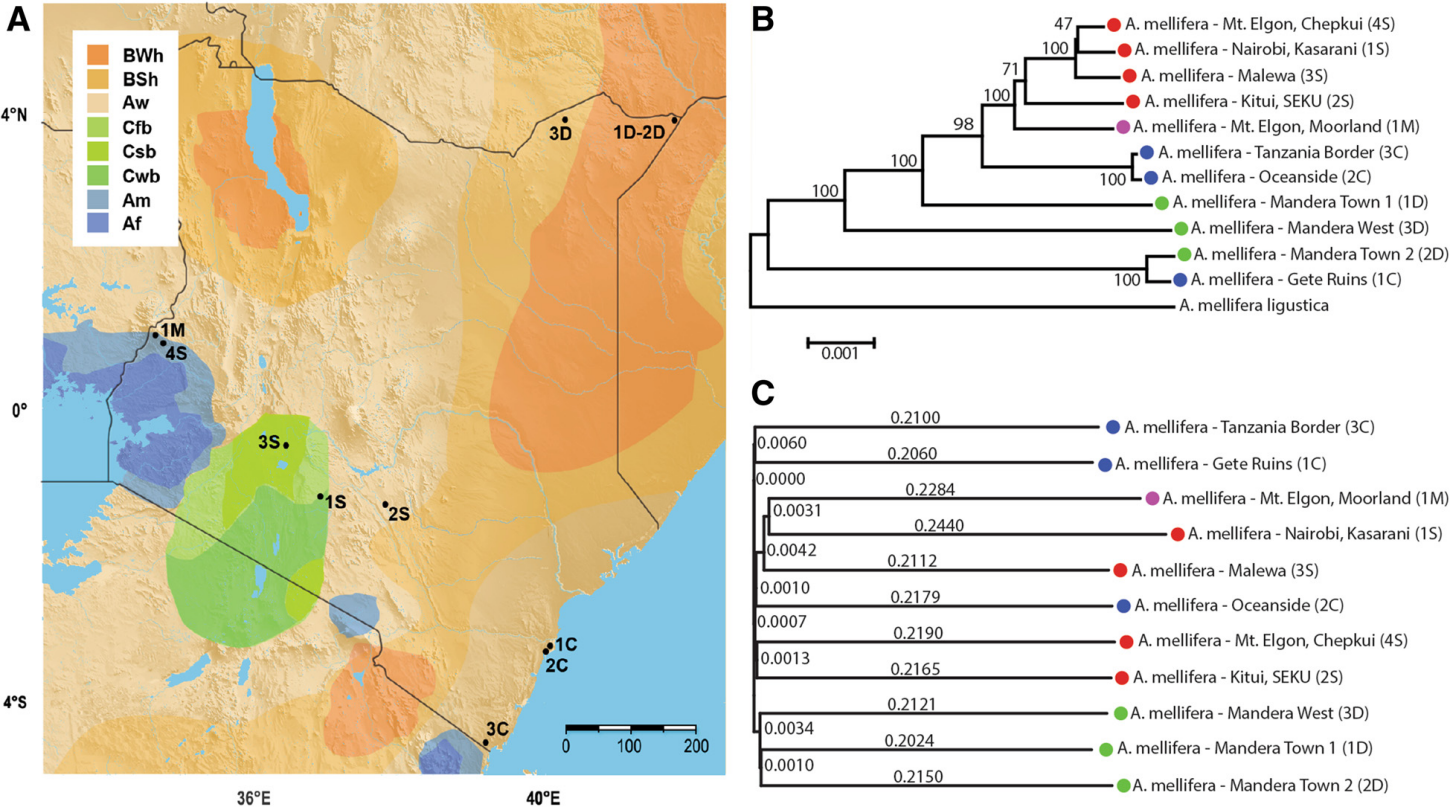
Geographic, phylogenetic, and principal component relationship of Kenyan honeybees. **a**) Geographic locations of sampled apiaries. Each individual is designated according to the ecological region in which it was sampled, with S = savannah, C = coast, D = desert, and M = mountains. Colors indicate Köppen-Geiger climate regions, see (Peel, et al. 2007) [106]. **b**) Neighbor-joining tree inferred from full mitochondrial sequences. The evolutionary distances were computed using the Maximum Composite Likelihood method and are in the units of the number of base substitutions per site. The tree is drawn to scale. **c**) Neighbor-joining tree inferred from full nuclear sequences. Numbers indicate the branch lengths

We sequenced the genomes of 11 individual worker bees from 11 different apiaries distributed throughout four distinct ecological regions in Kenya (savannah, coast, desert, mountain; see Fig. 1a for a map of the locations of the sampled individuals and Table 1 for details on the specimens). These regions have been previously described as areas of subspecies endemism [7, 9, 11]. Previous analyses with commonly used mitochondrial markers indicated that bees in these apiaries represented five different subspecies of *Apis mellifera*: *scutellata*, *monticola*, *littorea*, and *yemenitica* or *simensis* (see Additional file 1: Table S1 for further information about the precise sampling locations, and Muli et al. (2014) [12]. Sites S1, S2, S3 and S4 are within the described distribution of *Apis mellifera scutellata*. The original description of this habitat is “thorn woodland and tall grass savannah”, but *A. m. scutellata* is found in more diverse habitats across eastern Africa [7]. Sites 1C, 2C, and 3C are located in tropical coastal habitat where *Apis mellifera litorea* is said to occur. The apiary (1C) at the Gete Ruins is in the Arabuko Sokoke Forest Reserve, a remnant of the coastal tropical forest that supports a number of unique endemic species. Outside of this region, along the coast, there has been extensive alteration of the native habitat and potential incursion of *A. m. scutellata*. Sites 1D, 2D and 3D are located in the hot, dry Mandera district. At least two subspecies likely occur in this region, *A. m. yemenitica* and *A. m. simensis* [7, 9, 11]. The final subspecies, *A.m. monticola,* has received considerable attention in the literature (see Ruttner 1987, Hepburn and Radloff 1998, Gruber et al 2013 [7, 9, 13]). It has been described as “the bee of the rain forests of the East African mountains found at altitudes of 2000-3000 m” [7]. Only one site (1 M, Mt Elgon, Moorland) in our study was above 2000 m where *A. m. monticola* is said to occur. The bees were sampled during a 2010 survey of the health of Kenyan bee populations (see Muli et al. 2014 [12]). Several parasites and pathogens were identified in Kenyan bee colonies during this survey: Acute bee paralysis virus (ABPV), Black queen cell virus, (BQCV), Deformed wing virus (DWV), *Nosema apis* (a microsporidian gut parasite), and *Varroa destructor* (a parasitic mite); see Table 1 and Additional file 1: Table S1 for a listing of the parasites and pathogens found at each site. Notably, the honey bee colonies sampled at 1D, 2D, and 3D were free of all tested parasites and pathogens (see Table 1).

Across these 11 individuals, we identified ~3.6 million putative SNPs in the nuclear genome after filtering by quality score (see Additional file 2: Figure S1 for the frequency spectrum of alternative and reference calls). Using this set of variable sites, we first examined the evolutionary relationships among our sampled Kenyan honey bees through a phylogenetic analysis of both the mitochondrial and nuclear genomes (labeled mtDNA and nuDNA respectively). A phylogenetic analysis using the full mtDNA genome sequence (Fig. 1b) distinguishes between bees sampled from the savannah, coastal, and desert. The four savannah specimens (1S, 2S, 3S, 4S) all group together. Two of the three coastal bee specimens (2C, 3C) form an independent cluster. The three desert specimens (1D, 2D, 3D) are most distantly related to the savannah specimens, and could be either *A.m. yemenitica* or the recently described *A. m. simensis* [11]. Interestingly, one of the coastal bees (1C) groups with the desert bees. As mentioned previously, this coastal bee was collected from a remnant of the East African coastal tropical forest, and may represent a population that was originally in contact with the desert bee population. The single specimen from a mountain region (1 M) groups together with the savannah bees. Previous studies from Arias and Sheppard (1996) [10] used part of the NADH dehydrogenase subunit 2 (ND2) and the isoleucine transfer RNA (tRNA ILE) mtDNA region for phylogenetic analysis of the African honey bee subspecies. The corresponding sequence from 1 M is identical to that of Arias and Sheppard’s *A. m. monticola* bee (MONTIC 1). The nuDNA phylogeny distinguishes the desert individuals from a broad goruping of bees collected from southern mountain, coastal and savannah apiaries (Fig. 1c).

There are differences in both branch length and topology between the phylogenies constructed from the nuclear and mitochondrial genomes. Similar patterns of mito-nuclear discordance have been observed in other animal systems [48, 49]. Distinguishing which type of discordance is occuring is often difficult, but our results suggest biogeographic discordance, although we cannot completely rule out stochastic processes such as incomplete lineage sorting [50]. As described earlier, the mitochondrial tree (Fig. 1b) is roughly consistent with the geographic distribution (Fig. 1a) of the representative individuals and all savannah bees cluster together. The nuDNA-based tree however, distinguishes the desert bees from a broad southern population suggesting gene flow among the coastal, mountain and savannah bee populations (Fig. 1c). Moreover, there is no indication of stepwise ecoclines as described in Ruttner (1987) [7], and the northern desert bees form a distinct cluster, against the suggestion of Kerr [9]. The discordance between the mitochondrial and nuclear trees could be the result of limited dipersal of honey bee queens and movement of males over a larger geographic area. Unmated honey bee queens fly to male congregation sites that are delimited by topographic features and air flow turbulence. Upon mating with multiple males the females will leave the congregation site and found a colony. If female honey bees show natal site fidelity or limited dispersal similar to what has been found for the bumble bee *Bombus vosnesenskii,* the mitochondrial lineage would show biogeographical patterns [51]. If males come to congregation sites from a wide area then there would be greater gene flow at the level of the nuclear genome (see Hepburn & Randolff 1998 for a description of African honeybee mating [9]). The northern desert bee population may be relatively isolated given the arid climate (Fig. 1a, BWh) separting these populations from the south.

One of the tests for adaptive evolution that we wanted to apply uses an estimate of genetic differentiation, *F*_ST_, for two disjoint “populations” of individuals. We believed an appropriate choice would be to group individuals in geographically distinct populations and in accordance with the traditional definition of subspecies. One population consists of 1D, 2D and 3D, which we call the “Desert population”, and the other contains 2S, 3S, 4S, 2C and 3C, which we call the “Savannah population”. We used the “Overall *F*_*ST*_” tool on Galaxy to compare their *F*_ST_ with the *F*_ST_ for 1000 randomly generated choices of a set with three individuals and a disjoint set of five individuals (using Reich’s *F*_ST_ estimator; [33]). Overall, *F*_*ST*_ was estimated to be 0.02183 between these two groupings. Only 24 of the 1000 pairs had an equal or larger *F*_ST_, indicating that the probability of such a high score occurring by chance is *p* < 0.05 (see Additional file 2: for a detailed description of the global properties of *F*_ST_ between populations and comparisons to observations of Zayed and Whitfield 2008 [25]). In addition, using the “PCA” tool on Galaxy we performed a principal components analysis (PCA) of the individuals in our two defined populations, which further supports genetic differentiation between the Desert and Savannah populations (Additional file 2: Figure S2).

### Signals of adaptive evolution

**Fig. 2:**
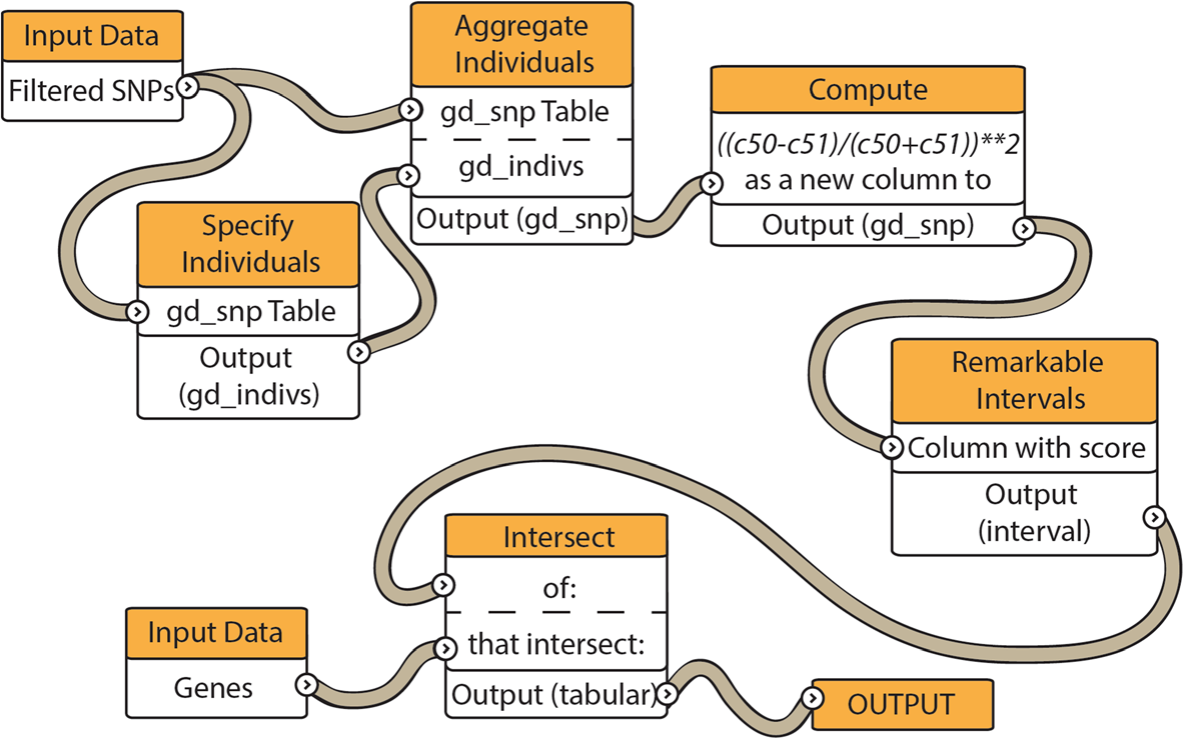
Example of a workflow using Galaxy commands. The workflow depicts the commands and tools used to carry out the test for genes located in a “run of homozygosity” (ROH; see the section detailing Test 4). The workflows and command histories for each test are available at https://usegalaxy.org/r/kenyanbee

We applied six tests (Table 2) to scan the genome for genes or genomic intervals showing signs of positive selection (adaptive evolution). Each test is available as a tool or reproducible workflow on the Galaxy website, http://usegalaxy.org/r/kenyanbee. Three tests are limited to SNPs observed in protein-coding regions, while three consider all polymorphic sites across the genome (see Table 2 for details). In broad terms, the protein-based tests look for a high proportion of amino acid-altering (and hence potentially function-altering) differences compared to the “silent” (amino acid preserving, and hence likely neutral) differences, and they can potentially detect selection over a wide range of evolutionary times. Thus, the protein-based tests include all Kenyan bees in our sample and may detect selection not limited to local geographic subpopulations. The tests of allele frequencies that include non-coding regions can in theory detect recent selection, presumably affecting only some individuals. The tools and workflows (Fig. 2) used to estimate the numbers of fixed differences form the reference genome, McDonald-Kreitman scores, runs of homozygosity (ROHs) and Tajima’s *D* values were specifically developed for the purpose of this study and are unreported in the initial description of the Galaxy tools to study genome diversity [6]. Most of the annotated genes that were identified by these tests are uncharacterized, and tests that considered genome-wide SNPs frequently found regions that do not intersect any annotated genes; however, for each test we describe a well-characterized gene that the test identified (see Table 2). We chose cutoff values for each of the protein-based tests to highlight examples of genes with large scores using tools available on the Galaxy platform. Future studies with a larger sample size will standardize the false discovery rate across each test for positive selection. For selected candidate genes (*Api m 6, FMRFamide receptor, NADH dehydrogenase*) where it was possible to design PCR primers, we validated the SNP calls detected from high throughput sequencing with Sanger sequencing. Because Sanger sequencing was performed on the coding regions of three selected candidate genes, not enough sites were validated to accurately estimate false positive and negative rates for the entire data set.

**Table 2:** Descriptions of statistical genomic analyses used

Test Number	Common name	Brief description	Region examined	Protein	Gene
1	pN/pS	nonsynonymous vs synonymous differences within Kenyan bees	Protein-coding	Venom allergen Api m 6	GB45615
2	—	nonsynonymous differences between Kenyan and European *Apis mellifera*	Protein-coding	FMRFamide receptor	GB51916
3	McDonald-Kreitman test	nonsynonymous differences between Kenyan bees and *Apis florea*	Protein-coding	NADH dehydrogenase (ubiquinone) 1 beta subcomplex	GB51330
4	ROH	runs of homozygosity	Genome-wide	FoxO	GB48301
5	*F*_ST_	differences between two populations of Kenyan bees	Genome-wide	Neuroligin-3	GB42603
6	Tajima’s D	allele frequency spectrum	Genome-wide	RpA70, ZFYVE26	GB44421 GB44416

### Test 1: pN/pS

Polymorphisms that alter the amino acid encoded by a codon are called nonsynonymous, while those that do not are called synonymous, or “silent”. Under neutrality, the majority of nonsynonymous mutations are expected to be deleterious and removed through the action of puryifying selection [52, 53]. Different modes of natural selection, however, can cause the frequencies of particular nonsynonymous mutations to increase within a population [54]. Hence, by comparing the rate of nonysnonymous mutations to the rate of synonymous mutations, which are not expected to be strongly affected by selection, insight can be gained into the selective constraints of particular protein coding regions. A common statistic used to infer the type and magnitude of selection acting on a given sequence is known as dN/dS, which simply divides the frequency of nonsynonymous differences per nonsynonymous site by the frequency of synonymous differences per synonymous site [39].

To investigate selection within a single species, this ratio is denoted as pN/pS because the differences between sequences are polymorphisms segregating within a population, not fixed differences between lineages. Diversifying, balancing or positive slection can be inferred when pN/pS > 1, while purifying selection is suggested when pN/pS < 1 [55–60]. It is important to be aware that interpretations of pN/pS may be complicated by the presence of multiple alleles or recently diverged sequences when analyzing within-species data. Recent theoretical and empirical work using modifications from the codon based phylogenetic substitution model of Goldman & Yang (1994) [61] to estimate dN/dS has demonstrated that in certain cases of positive selection, dN/dS < 1 when calculated from within-species data [62, 63]. Here, we estimated pN/pS across the genome for our Kenyan samples using the heuristic counting method of Nei & Gojobori (1986) [39].

For each of the 15,314 annotated proteins, we partitioned coding-region SNPs into non-synonymous and synonymous, which permits pN/pS to be calculated for proteins with at least one synonymous SNP (see Additional file 1: Table S4A for a list of genes with pN/*p*S ≥ 2 and Additional file 1: Table S4B for the results of a GO analysis). Several genes found to have high pN/pS are suggested to be under selection in other species, including GB52930 (the honey bee ortholog of *Drosophila* gene *armitage*) which appears to be under selection in *Drosophila simulans*. Furthermore, GB48792 (the ortholog of *Drosophila* gene *dunce*), associated with learning and memory, has been identified as one of the rapidly evolving genes in primitively eusocial bees [64, 65].

One gene found to have a high pN/pS is venom allergen *Api m 6* (GB45615), with 8 nonsynonymous and 1 synonymous mutations, for which pN/pS was estimated to be 2.48. Kettner, et al. (2001) [66] isolated this component of honeybee venom and observed four protein isoforms, which Peiren, et al. [67] attribute to a high level of polymorphism at a single genomic locus. Importantly, and somewhat disturbingly, our scan reported the gene in two genomic locations (GB45615 in Group16.6, and GB49510 in GroupUn3525), both with high pN/pS. Peiren, et al. (2006) [67] had observed this situation with an earlier *A. mellifera* genome assembly, and concluded that a high level of polymorphism within and around the gene caused the assembly algorithm to erroneously separate the two haplotypes. We concurred with their analysis and sought to determine the correct numbers of nonsynonymous differences in this gene.

By decreasing the gap penalty between the two haplotypes, we created a single alignment of the reference region around gene GB45615 that included the reads formerly aligned to GB49510 to analyze the insertions and deletions. SAMtools reported 7 nonsynonymous substitutions, i.e., one of the previously reported differences was lost [67]. In an interval containing the gene and its immediate flanking regions (positions 5100 to 6100 of Group16.6), we detected 10 short insertion/deletions, including a one-codon deletion (relative to the European reference sequence) in the second exon, and a two-codon insertion immediately before the gene’s last (non-stop) codon, at a position where Peiren et al. (2006) [67] also observed a two-codon insertion (see their Fig. 1c).

We designed primers (Additional file 1: Table S3) to amplify the *Api m 6* region in our Kenyan samples as well as in two individuals each of *A. m. carnica* and *A. m. ligustica* ancestry derived from US bee populations. Using Sanger sequencing, we confirmed the 2 nonsynonymous polymorphisms contained in exon 1, the 2 nonsynonymous polymorphisms present in exon 2 as well as the 3 nonsynonymous polymorphisms located across exon 3. Furthermore, we were able to confirm the two-codon insertion relative to the reference before the gene’s last non-stop codon within the Kenyan samples (see Additional file 3: Alignment S1).

Although the pN/pS ratio can provide insight into the selective constraints of a genomic region, we cannot exclude the potential influence of demography, population structure and biased allele frequencies on our results. However for the case of *Api m 6,* it is unlikely that population structure strongly affects our conclusions. Between the Savannah and Desert subpopulations, *F*_*ST*_ over the locus is only .00836 and neither form monophyletic clades in the phylogeny obtained from the region (Additional file 2: Figure S3). It is also unlikely that incorrect gene models have inflated pN/pS values due to the misclassifcation of sites. The Amel_4.5 assembly gene annotation (OGSv3.2) we used here is a significant improvement from the original annotation (OGSv1.0) contained in the previous assembly. In the OGSv3.2 annotation, gene models were predicted *de novo* and inferred from RNAseq and protein data [38]. Furthermore, we recognize that pN/pS can be artificially inflated if a gene contains a small number of polymorphic sites and mutations occur at nonsynonymous sites more frequently by chance. For genes with pN/pS ≥ 2, we consider this effect unlikely to impact our results, as the number of polymorphic sites per gene range from 7 to 50.

### Test 2: Fixed differences from the (European) reference genome

We looked for genes with an unusually large number of fixed amino acid differences between our Kenyan samples and the *Apis mellifera* reference genome, which is of European ancestry [24]. This property may indicate adaptive evolution in one or both lineages since the separation of the European and African honey bees. Although similar in nature to other tests, such as Mcdonald-Kreitman (discussed later), here we are specifically searching for sites that differ from the reference genome and have risen to complete fixation within the Kenyan samples. Because we are only interested in genes that contain amino acid differences that have arisen to complete fixation, this test may not detect genes with large numbers of high frequency nonsynonymous polymorphisms that could be detected by other methods, such as the Mcdonald-Kreitman test. Of the SNPs reported on Galaxy, 290,445 are fixed in the 11 samples with an allele differing from the reference genome. These SNPs should be treated with some caution because erroneous nucleotides in the reference sequence are highly likely to appear in this list.

Using Galaxy commands (available in a workflow developed at http://usegalaxy.org/r/kenyanbee), we found that 4,934 of those SNPs create a putative amino acid substitution, with 3,242 affected genes. Similarly we found 8,628 synonymous coding-region differences among the 290,445 SNPs, distributed among 4,638 genes. Among all genes with a fixed difference, there was an average of 1.86 fixed synonymous differences and 1.52 fixed nonsynonymous differences. We then searched for genes where the frequency of fixed amino acid differences from the European reference (E_N_) exceeds the number of amino acids that are polymorphic within Kenyan bees (P_N_). We sorted genes by the ratio E_N_/(P_N_ + 1) (1 is added in the denominator in order to include genes with no polymorphism; genes with a ratio of at least 2.0 are given in Additional file 1: Table S5). Note that GO analysis was not performed because only a small number of genes was associated with a FlyBase annotation and DAVID identifiers.

**Fig. 3:**
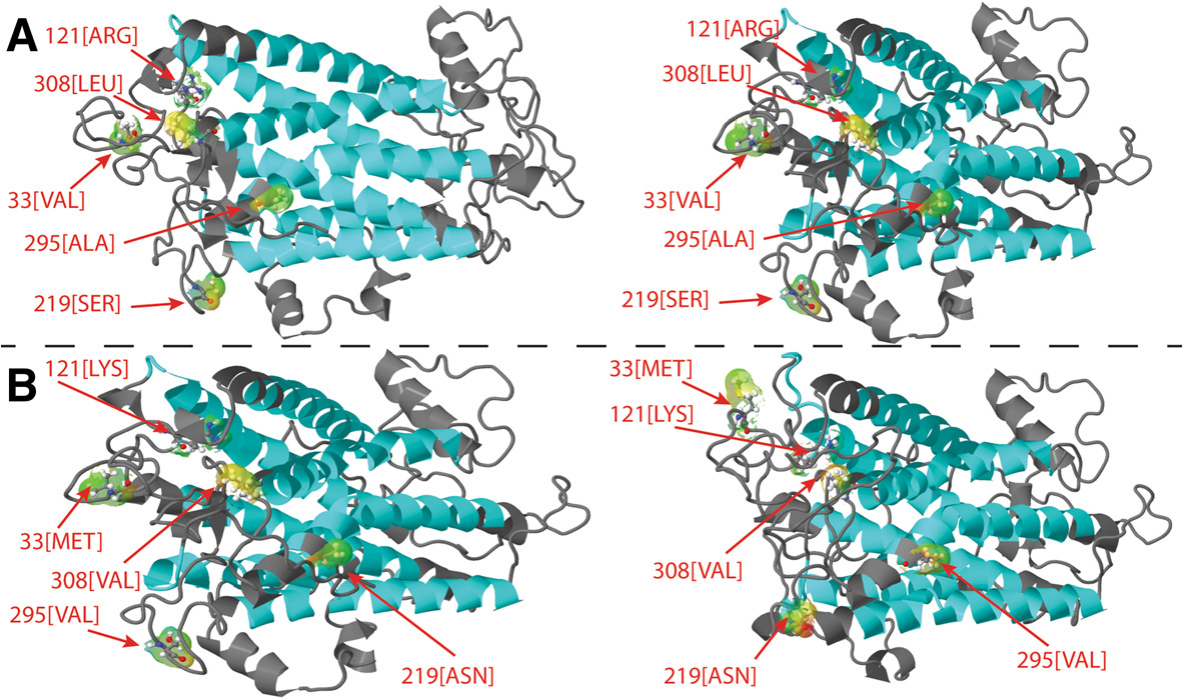
Predicted FMRFamide receptor structures in European **a** and Kenyan **b** bees. Five residues in the extracellular domains (gray) were found to differ at positions 33, 121, 219, 295, and 308 between these two populations. The configuration of the extracellular domains is altered despite the fact that the mutations are not drastic. The transmembrane regions (cyan) follow the homologues positions of *D. meloganogaster* studied by Merte and Nichols (2002) [107]. Red labels represent the position and amino acid of the structure altering mutation

A gene tied for the second highest score was the *FMRFamide receptor* (GB51916; Fig. 3). FMRFamides are small molecules with neuropeptide-associated activity that play critical roles in several invertebrate physiological processes such as vision, reproduction, feeding and circulatory system regulation [68]. Since this gene was independently cloned and sequenced (GenBank Accession Number ACI90286), rather than a computational prediction based on the genome assembly, we could confirm the assembly’s accuracy in this region. The gene contains 5 nonsynonymous differences between the reference and the Kenyan samples, along with 2 synonymous differences. Within the Kenyan samples, 8 variant nucleotides were called, all synonymous. These observations are consistent with a gene that has undergone positive selection in one of the lineages leading from the split of the Kenyan and European populations. It is also possible that these amino acid substitutions could be a result of genetic drift and we cannot exclude its influence. These amino acid differences are predicted to lead to significant changes in protein structure. Specifically, the extracellular domains differ between the Kenyan and European protein structures (Fig. 3).

The analysis was complicated by the presence of an assembly gap in the honeybee reference sequence (indicated by a run of the letter N) in the FMRFamide receptor’s last (i.e., fourth) exon, namely positions 498032-498531 of the sequence called Group3.3. We substituted the corresponding sequence from ACI90286 for the run of Ns and realigned with our sequence data. No new SNPs were predicted. However, manual inspection of the alignments suggested that one of the automatic SNP calls of a synonymous difference among the Kenyan samples might be a false positive, but did not materially affect our conclusions. We designed primers (Additional file 1: Table S3) to amplify across exon 2 and exon 3 of the gene and through Sanger sequencing we confirmed the presence of the 3 fixed nonsynonymous differences between the European reference and our Kenyan samples present in this region (see Additional file 4: Alignment S2).

### Test 3: McDonald-Kreitman test

Comparing the pattern of nonsynonymous and synonymous polymorphism within a species to the pattern of nonsynonymous and synoymous divergence between species can potentially identify positive selection operating over long evolutionary time periods [69]. McDonald and Kreitman (1991) [70] hypothesized that regions containing an excess of nonsynonymous to synonymous divergence relative to the nonsynonymous to synonymous polymorphism may suggest positive selection and adaptive evolution. For a given gene, the McDonald-Kreitman test constructs a contingency table from the number of synonymous subsitutions (D_S_), the number of nonsynonymous substitutions (D_N_), the number of synonymous polymorphisms (P_S_) and the number of nonsynonymous polymorphisms (P_N_). Note P_S_ and P_N_ refer to the actual counts of substitutions, and differ from the rates pN and pS used in Test 1. Genes where the ratio of (D_N_/D_S_)/(P_N_/P_S_) exceeds 1 may be where purifying selection has not driven advantageous alleles to fixation within the species, but has done so over the longer evolutionary time separating the species. Similar to Shapiro et al. (2007) [71], we calculated this ratio, termed the Fixation Index (FI), for each annotated gene along the genome.

For the second species used in this test, we chose the dwarf honeybee, *Apis florea*, for which unassembled sequence is available in GenBank. A table that reports the computed FI scores for each gene is available at the Galaxy web server (https://usegalaxy.org/r/kenyanbee). In total, we identified 810,794 putative inter-species nucleotide differences. We note that high FI scores could be the result of a poor alignment with the outgroup sequence. For individual candidate genes, we manually inspected the *A. florea* alignment and did not find any unusual patterns (see Additional file 1: Table S6A for a list of genes with FI scores ≥ 10 and Additional file 1: Table S6B for the results of a GO analysis).

When we simply sorted the table by decreasing FI score, the top genes were surprisingly dominated by cases where the computed pN/pS was extremely small, rather than cases where the nonsynonymous/synonymous ratio for interspecies variants was unusually high. For genes where we were able to estimate pN/pS, the average value is 0.215, but genes with the highest computed FI score typically had pN/pS around 0.01. Among genes with pN/pS ≥ 0.2, the one with highest FI score is that for *NADH dehydrogenase (ubiquinone) 1 beta subcomplex, subunit 2* (*NDUFB2*, GB51330). We observed 1 nonsynonymous and 1 synonymous differences in our *A. mellifera* data (pN/pS = 0.237), but 17 nonsynonymous and 1 synonymous interspecies difference, for an FI score of 17. The 1 nonsynonymous and 1 synonymous difference found with the Kenyan individuals were confirmed to be located in the first exon through Sanger sequencing (see Additional file 5: Alignment S3). In a situation like this, where the gene appears to be quite well conserved within the species but quite divergent from a related species, one needs to consider the possibility that the genes being compared are paralogs not orthologs (i.e., they diverged because of a duplication event in a common ancestor of the two species). We consider that to be quite unlikely for this gene, because *A. mellifera* and *A. florea* genes are embedded in assembled sequences that align for over a megabase without interruption. A second interesting gene in this group, GB48492 (*juvenile hormone binding protein 1, JHBP-1)*, is associated with adult feeding behavior and is the ortholog of *Drosophila takeout* (*to*). This gene encodes juvenile hormone binding protein and in *Drosophila* is associated with circadian rhythm (reviewed in Sokolowski, et al. (2001) [72]) and behavioral responses to starvation [73, 74]. In honey bees, *JHBP-1* might be under selection allowing adaptation to the vastly different environments these bees inhabit, possibly leading to differences in adult worker development and responses to food availability and different food resources.

### Test 4: Runs of homozygosity

As an allele rises in frequency within a population, nearby variants can also rise in frequency, transforming the usual patterns of genetic diversity for that region and creating stretches of high homozygosity. Thus, stretches of the genome containing reduced allelic diversity are potential signs of a selective sweep and can be indicative of recent positive selection [75]. Significant selective sweeps within a population can result in extended tracts of homozygous polymorphisms, known as runs of homozygosity (ROH) [76, 77]. Here, we compared allele frequencies between Savannah and Desert individuals to identify ROHs in either population. Using tools on the Galaxy server (available in a workflow developed at http://usegalaxy.org/r/kenyanbee), allele frequencies were estimated for each population and the genome was scanned for intervals containing consecutive homozygous genotypes. Furthermore, we performed 1000 randomizations of the data, so that any interval reported had a probability less than 0.001 of being discovered purely by chance. We then intersected these intervals with the coordinates of annotated genes to create population-specific lists of candidate genes containing ROHs (see Additional file 1: Table S7 for a list of genes).

**Fig. 4:**
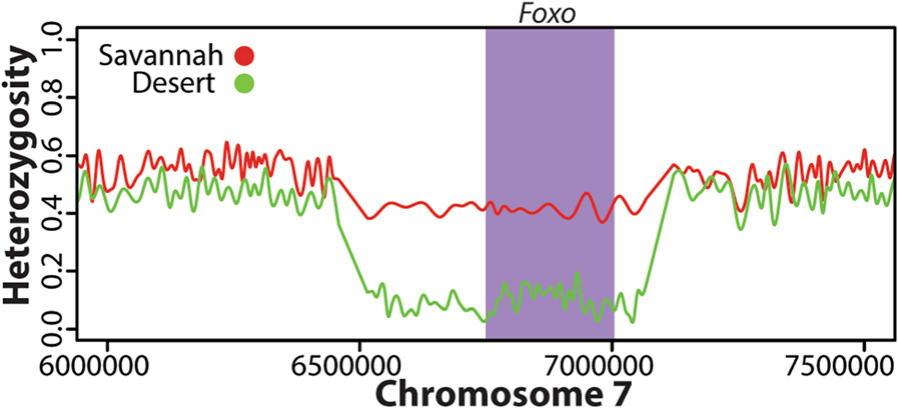
Evidence for a selective sweep near Forkhead Box Protein O (*Foxo)* in Desert population*.* The average heterozygosity is shown for all individuals sequenced in the Savannah and Desert populations across a 1.5 Mb region on chromosome 7. The shaded area indicates the location of *Foxo.* A marked reduction in heterozygosity for the region surrounding *Foxo* is observed in the Desert population

We observed one such region surrounding the Forkhead Box Protein O (*Foxo,* GB48301) gene in the Desert population (Fig. 4). Heterozygosity levels in the Desert population are consistent with the Savannah bees around 500 kb upstream and downstream of the region. However, heterozygosity levels are strongly reduced in a 500 kb stretch containing *Foxo* within the Desert population relative to the Savannah population. The protein expressed by *Foxo* is a transcription factor that is thought to play a major role in caste differentiation and division of labor by regulating insulin signaling [78, 79]. *Foxo* was also found in a QTL that suppressed reproduction of *Varroa destructor*, a major parasite of honey bees [80]. Interestingly, a recent survey of honey bee populations in Kenya revealed that *Varroa destructo*r is present throughout the southern and central honey bee populations but absent from the northern desert regions [12]. However, *Varroa* was likely introduced relatively recently (ie, with the last 10 years) into Kenya [81] and thus likely does not account for this population difference.

Besides selective forces, ROHs can also result from high levels of inbreeding or appear as an artifact of population history [82–84]. In our sample, inbreeding is not expected to have a major effect as all individuals were collected from wild populations where queens mate with an average of 12 males and mating occurs between colonies over a several kilometer radius. It is suggested the purpose of this polyandrous behavior is to avoid the accumulation of deleterious recessive mutations due to inbreeding as well as to increase genetic diversity [85]. Furthermore, studies of populations of African bees demonstrate that there is rapid colony turnover, with more than 80 % of the queens in a particular site being replaced by genetically unrelated queens [86]. We cannot exclude past demographic events or low statistical power resulting from our limited sample size as contributing factors to the ROHs we observe. However, the *Foxo* locus shows a signal consistent with recent positive selection in the Desert population and seems like a biological plausible candidate that future studies can provide further insight on.

### Test 5: F_ST_

Strong selective sweeps can create regions with differences in allele frequencies and increased genetic differentiation between populations [87]. Genomic intervals containing high levels of *F*_ST_ between two populations indicate regions potentially under positive selection in one or both lineages [45]. We identified several such genomic intervals displaying signatures of positive selection between the Desert and Savannah populations. First, at each SNP site we calculated the Reich-Patterson estimator of *F*_ST_ between the two populations (see Additional file 2: Figure S4 for the distribution of *F*_*ST*_). With a low-sample size, yet a large number of SNP markers, the Reich-Patterson method provides an unbiased and highly powerful estimate of *F*_ST_ [88]. Negative *F*_*ST*_ values are the result of higher genetic differentiation for individuals within a population than between populations. To locate regions of the genome with signatures of positive selection, we then searched for areas with dense concentrations of high scoring SNPs using the “Remarkable Intervals” tool on Galaxy. Furthermore, we performed 1000 randomizations of the data, so that any interval reported had a probability less than 0.001 of being discovered purely by chance. We located 184 such intervals with an average length of 3,633 bp. 112 annotated genes were found in the highest scoring intervals (see Additional file 1: Table S8A for a list of genes and Additional file 1: Table S8B for the results of a GO analysis). One of the genes located in an identified interval was GB46429 (ortholog of *Drosophila* gene *ebony*), which is involved in regulation of cuticle sclerotization and pigmentation in *Drosophila* [89, 90]. Since insect cuticle functions as the first line of defense against environmental stressors, it is possible that in African honey bees these differences represent an adaptation to different climatic and ecological conditions found between the Desert and Savannah regions. Indeed, this gene was also found to be under selection in African *Drosophila melanogaster* as a likely adaptation to differences in altitude [91]. Additionally, *Foxo*, identified previously in the ROH test, was found to be located within a high scoring interval.

One of the genes identified with our *F*_*ST*_ test is GB42500, an ortholog of the *Drosophila* Peptidoglycan recognition protein-LC (PGRP-LC). Similarly, Viljakainen et al. (2009) [92] found signatures of positive selection for a PGRP gene in an ant genus *Myrmica*. We identified several other immune genes including *relish* (GB55264) which is implicated in honey bee immune response [93, 94]. Interestingly, previous studies have indicated that the Savannah bee populations frequently are infected with two common European viruses (Black queen cell virus and Deformed wing virus) while the Desert bees were virus-free (see Table 1 and Additional file 1: Table S1), however, it is unclear if this difference is due to variability in immune competence or exposure [12].

**Fig. 5:**
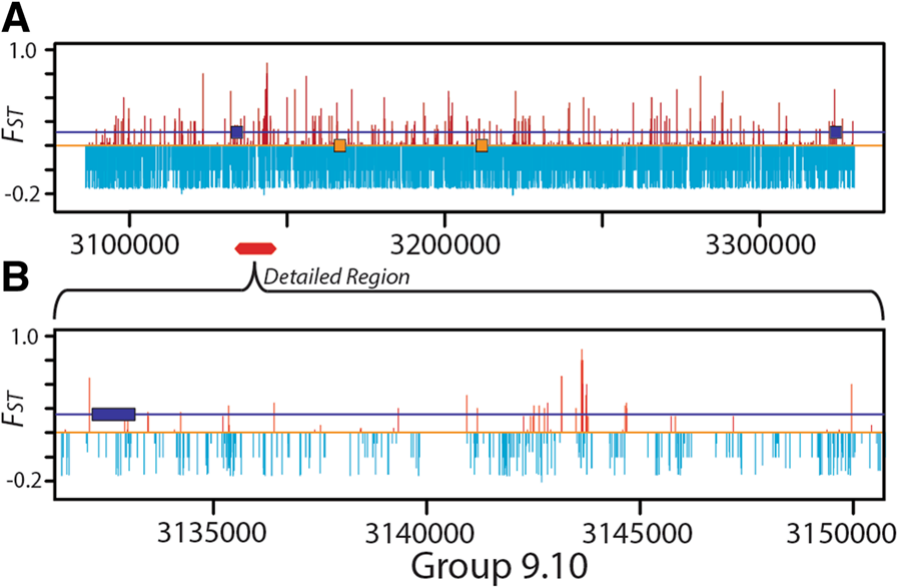
*F*_ST_ analysis of the chromosomal region containing the *Neuroligin-3* gene. **a**) Plot of *F*_ST_ (Reich-Patterson formulation) between Desert and Plains bees at 4,507 SNPs in a 200-kb interval on Chromosome 9 (Group9.10) that spans the first two exons of the Neuroligin-3 gene (GB42603; blue rectangles; transcribed right-to-left). Orange rectangles indicate the two exons of annotated gene GB42882, putatively transcribed on the opposite strand, though we find the evidence for existence of a functional gene unconvincing. Genome-wide, 10 % of the *F*_ST_ values are larger than 0.20 — indicated with red. Light blue bars represent non-significant *F*_*ST*_ values. **b**) The interval 3,143,154-3,143,747 has a statistically significant concentration of SNPs with *F*_ST_ in the highest 10 %, meeting our criterion for putative positive selection by the *F*_ST_ criterion

Another gene of interest is *Neuroligin 3* (*Nlg3*, GB42603; Fig. 5). *Nlg3* is involved in central nervous system development and is expressed in higher order processing centers in the honey bee brain [95]. The interval with high *F*_ST_ that intersects *Nlg3* spans around 600 bp with the 202 kb intron between exon 1 and exon 2 (Fig. 5b). *Nlg3* has several orthologs found within the insect class that also have long first intron sequences, including the parasitoid wasp (*Nasonia vitrpennis*, 130 kb), the red flour beetle (*Tribolium castaneum*, 100 kb), the dwarf honeybee (*Apis florea*, 202 kb), the leafcutter bee (*Megachile rotundata*, 95 kb), the pea aphid (*Acrythosiphon pisum,* 143 kb) and the bumble bee (*Bombus terrestris*, 187 kb). The evolutionary conservation of a large intronic region hints at the presence of essential regulatory elements.

### Test 6: Tajima’s D

Selective sweeps often cause distortions in the observed allele frequency spectrum at a given genomic region [96–98]. Genetic variability is greatly reduced in the area where a selective sweep has taken place, however, gradually over time new mutations appear. These mutations initially occur at low frequencies, creating regions with an excess of rare polymorphic variants and deficiencies of common variants [42]. Several methods exist for detecting these regions where the frequency spectrum significantly deviates from neutral expectations, such as Tajima’s *D* statistic [40]. We calculated *D* on a genome-wide level using 5 kb, non-overlapping, sliding windows to discover genomic regions containing extended stretches of an excess of rare polymorphisms relative to the total number of segregating sites, indicating positive selection. However, it is important to note that values of Tajima’s *D* can be affected by population structure, demographic history and recombination [99]. Negative *D* values may be caused from an excess of rare variants as a result of a recent population growth [100]. In our sample, values of *D* are slightly skewed towards negative values (Additional file 2: Figure S5).

To differentiate between selective processes and demographic effects we also complemented our sliding window estimation of *D* with another test, Fay and Wu’s *H,* that considers the amount of very high-frequency polymorphisms relative to intermediate-frequency ones, in addition to using information from an outgroup [42]. As suggested by Zeng et al. (2006) [41], we estimated the normalized version of the *H* statistic. For an outgroup, we mapped unassembled reads of the closely related Asiatic honeybee *A. cerana*, available from the NCBI Short Read Archive (SRX339508) to the *A. mellifera* reference genome following the same procedure as the experiment that originally generated the data [28]. We note that interpretations of *H* may be complicated by the presence of missing data in the *A. cerana* sequence.

**Fig. 6:**
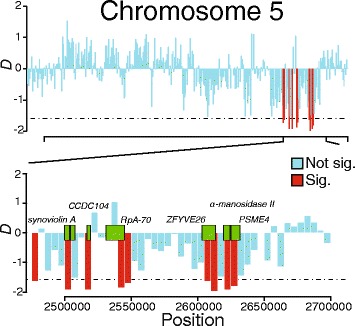
Selected chromosomal regions with signatures of positive selection according to Tajima’s *D* analysis. The top panel shows a section of chromosome 5. Tajima’s *D* was calculated in 5 kb windows and scores are represented by vertical bars. Any bar colored red represents a window where Tajima’s *D* is significantly negative. Conversely, bars colored light blue represent windows where Tajima’s *D* is not significantly different from values expected under a site evolving neutrally. The bottom panel is a magnification of the area containing a ~20 kb stretch of significant Tajima’s *D* scores. Green blocks represent locations of genes contained within the interval

When tests against a neutral null hypothesis, such as *D* and *H*, are performed repeatedly across a genome in a sliding window analysis, an issue of multiple comparisons arises. Although several strategies have been proposed to account for multiple testing, such as controlling the false discovery rate or Bonferroni procedures, here we use the experiment-wide simulation approach suggested by Nielsen et al. (2005) [43]. Several genes were found in intervals testing significantly negative for both Tajima’s *D* and Fay and Wu’s *H* (see Additional file 1: Table S9A for a complete list and Additional file 1: S9B for the results of a GO analysis), including *Derlin-1* (GB46979). Derlins are rhomboid pseudoproteases involved with endoplasmic reticulum associated degredation and play a role in the dislocation of misfolded proteins [101]. A ~20 kb region of chromosome 5 harbors several genes that intersect significantly negative windows of Tajima’s *D* (Fig. 6). Furthermore, Fay and Wu’s *H* values in the region are also detected as significantly negative (Additional file 1: Table S9A), providing additional evidence of a true departure from neutrality. Genes located in this region include *replication-protein- A 70 kDa subunit* (*RpA70*; GB44421), *zinc finger FYVE domain containing protein 26* (*ZFYVE26;* GB44416) and *alpha-mannosidase II* (GB44414).

Purifying selection may also result in significant Tajima’s *D* and shift the frequency spectrum towards low frequency variants [102, 103]. Nonsynonymous mutations are expected to be under stronger purifying selection than changes at synonymous or noncoding sites because they alter the encoded protein and may be deleterious. To examine the effect of purifying selection on the frequency spectrum, we estimated Tajima’s *D* genome-wide at three classes of sites: noncoding, synonymous and nonsynonymous. We observed a potential influence of purifying selection across the genome, as *D* is most negative at nonsynonymous sites (-1.07). At synonymous and noncoding sites, *D* was estimated as -0.61 and -0.32 respectively. Thus, because of the likely impact of purifying selection and unknown demographic effets, we caution against interpreting the genes detected by the test as being under positive selection.

## Conclusions

Here, we describe the expansion and demonstrate the use of a single, user-friendly interface on the Galaxy webserver to elucidate population structure and signatures of selection from full-genome sequence data of individuals sampled across different ecological regions. This platform allows for the efficient integration of several different analyses and tests, allowing researchers to examine selection over different evolutionary time periods and make their data publically available, facilitating transparency and reproducibility. In our study, we examined the population structure of 11 individual honey bees collected from throughout Kenya. Because of the large amount of genetic markers available and despite the relatively low number of samples, we were able to demonstrate that the sampled specimens segregating into two populations, with one population found in the northern desert region and the second spanning the savannah, coast and mountain. Both the analyses of the mtDNA and the nuclear DNA distinguished between these two main populations, though the grouping of three individuals were variable, suggesting that these specimens represent populations with complex histories. The two main populations are geographically separated by series of deserts that undoubtedly prevent migration and matings. The Savannah population spans southern and central Kenya and includes bees sampled from mountains, the coast, and the savannah. African bees can migrate long distances (potentially more than 100 km), thus facilitating gene flow across this large region [23]. The Desert and Savannah populations clearly experience distinct ecological and climatic conditions as well, and recent studies indicate that they are also experiencing different parasite and pathogen challenges [12].

Based on our analyses of genome-wide signatures of selection, there are several genes and gene regions that may have been influenced by selective forces at different evolutionary time scales. Many of these genes have been shown to play a role in metabolism/nutrition (*Foxo, NDUFB2, takeout*), neuroplasticity/neuronal development (*Neuroligin 3, RpA70, ZFYVE26*), immunity and parasite resistance (*Foxo, peptidoglycan recognition protein-LC, relish, helicase 25E, hemolectin*), reproduction (*armitage* and *dunce*), gland development and gland secretions (*Api M 6, MRJP1*, *thickveins)*, which may be involved in pheromone, brood food, and venom production and finally cuticle formation (*ebony*) and cuticular hydrocarbon synthesis (*fatty-acyl CoA reductase*) which are critical for protecting insects from environmental stressors, and, in the case of cuticular hydrocarbons, may also be involved in chemical communication. Overall, while the suites of genes identified in these different tests were essentially non-overlapping, there was overlap in the general processes they are associated with; genes involved in the basic processes of metabolism and cell differentiation were found to be regulated across multiple evolutionary time scales. Thus, selection may operate on different genes and on different time scales in these different honey bee populations, but these different selective pressures modify conserved pathways, as is the case for “genetic toolkits” identified in studies of evolutionary development biology [103, 104]. Although the tools and workflows developed on Galaxy provide researchers with a user-friendly and integrative platform to scan genomic data for signatures of selection, we also wish to emphasize that alternative explanations may be responsible for statistically significant results. We encourage users to utilize the background information provided in this study and refer to the original publications of each test to understand how results may be impacted by various factors such as demographic history, drift and population structure.

While we validated the results of these genome-wide tests for several selected genes, it is important to stress that whole-genome computational analyses need to be used with care. A search though millions of nucleotides or thousand of genes to maximize or minimize some quantitative feature will frequently identify cases where the validity of the feature breaks down because certain assumptions are violated, such as where the genome assembly or gene annotations are incorrect. Furthermore, genome scans can lead to spurious results in GO analysis because of the potentially large numbers of false positives, so we further reiterate the importance of experimental validation and aim to motivate detailed studies of individual candidate loci in the future [105]. Thus, at least until assembly and annotation methods become error-free, the results of high-throughput data collection and analyses often need careful investigation and/or experimental validation.

### Availability of supporting data

The data supporting the results of this study are available on the NCBI Short Read Archive, Accession Number: [SRP037570]. Supporting tables and workflow histories are available at https://usegalaxy.org/r/kenyanbee.

## Additional files

Additional file 1: **Supplementary tables (S1-S9).**

Additional file 2: **Supplementary material.**

Additional file 3: **Supplementary alignment 1 (Api M6).**

Additional file 4: **Supplementary alignment 2 (FMRFamide).**

Additional file 5: **Supplementary alignment 3 (NADH).**

## Abbreviations

ABPV: Acute bee paralysis virus
BQCV: Blackened queen cell virus
D_s_: Number of synonymous substitutions
D_N_: Number of nonsynonymous substitutions
DWV: Deformed wing virus
E_N_: Number of fixed amino acid differences from the European reference genome
FI: Fixation index
GO: Gene ontology
mtDNA: Mitochondrial genome
nuDNA: Nuclear genome
NCBI: National Center for Biotechnology Information
PCA: Principal components analysis
P_s_: Number of synonymous polymorphisms
P_N_: Number of nonsynonymous polymorphisms
pS: Rate of synonymous polymorphisms per synonymous site
pN: Rate of nonsynonymous polymorphisms per nonsynonymous site
RMS: Root mean square
ROH: Runs of homozygosity
SNP: Single nucleotide polymorphism
