# Supplementary material for: Genome-wide analysis of signatures of selection in populations of African honey bees (Apis mellifera) using new web-based tools
Source: BMC Genomics. 2015 Jul 10;16(1):518. doi: 10.1186/s12864-015-1712-0 (PMC4496815; doi:10.1186/s12864-015-1712-0)
Supplement: Supplementary file 2 — Supplementary material. [file 12864_2015_1712_MOESM2_ESM.docx]

**Supplementary Materials**

**Global properties of honey-bee *F*_ST_.** The large set of SNPs being made available can be used for a variety of purposes, which we illustrate here by attempting to recapitulate two observations about *F*_ST_ between other pairs of honey-bee populations reported by Zayed and Whitfield (2008). The observations were that (1) *F*_ST_ was higher in protein-coding regions than in non-coding regions and (2) *F*_ST_ was negatively correlated with G+C content.

We explored these hypotheses using the two populations discussed in the main paper, which we called the plains and desert individuals, and the 2.4 million high-quality SNPs. For the first hypothesis, we added a table of positions of the coding exons to Galaxy, and used Galaxy commands to average the *F*_ST_ values determined by three definitions: the original definition of Sewall Wright (Wright 1954), the popular Weir-Cockerham (1984) estimator, and the recent estimator of Reich and Patterson (Reich et al., 2010). The Reich-Patterson estimator has been shown to be the most accurate of the three when using many SNPs from a few individuals (Willing et al. 2012). For the two populations studied here, our results (Table S1) fail to reflect the first observation of Zayed and Whitfield, but instead are consistent with the expectation that allele frequencies will be more similar between populations in coding regions because the level of purifying selection is higher than in non-coding regions.

**Table SM1.** Average *F*_ST_ between the plains and desert honey bees, using three definitions of *F*_ST_.

| formula | coding | non-coding |
| --- | --- | --- |
| Wright | 0.078 | 0.079 |
| Weir-Cockerham | -0.0015 | 0.0002 |
| Reich-Patterson | 0.0046 | 0.0063 |

To explore the relationship between *F*_ST_ and G+C content, we added a table giving G+C content in non-overlapping 10kb windows across the honey bee genome. Galaxy commands then averaged the *F*_ST_ in the intersection of windows with coding and non-coding DNA, where the windows were stratified by G+C content. Table S2 shows the results for the Reich-Patterson estimator. Results for the other two formulations of *F*_ST_ also support the observations that, indeed, *F*_ST_ is negatively correlated with G+C content, for both coding and non-coding regions, but most of that effect comes from the relatively high *F*_ST_ in the 24% of the 10kb intervals (6,497 of 26,914) whose G+C content is under 25%.

The reader can find “workflows” (sets of commands) on Galaxy that generate the data for Tables S1 and S2, making it easy to reanalyze the data is different ways.

**Table SM2.** Average *F*_ST_ between the plains and desert honey bees, using the Reich-Patterson formulation, within 10kb windows where the percentage of G and C nucleotides is in a specified range.

|  | coding | non-coding |
| --- | --- | --- |
| G+C < 25% | 0.011 | 0.013 |
| 25% <= G+C < 30% | 0.0028 | 0.0063 |
| 30% <= G+C < 35% | 0.0021 | 0.0045 |
| 35% <= G+C < 40% | 0.000055 | 0.0045 |
| 40% <= G+C | 0.0022 | 0.0039 |


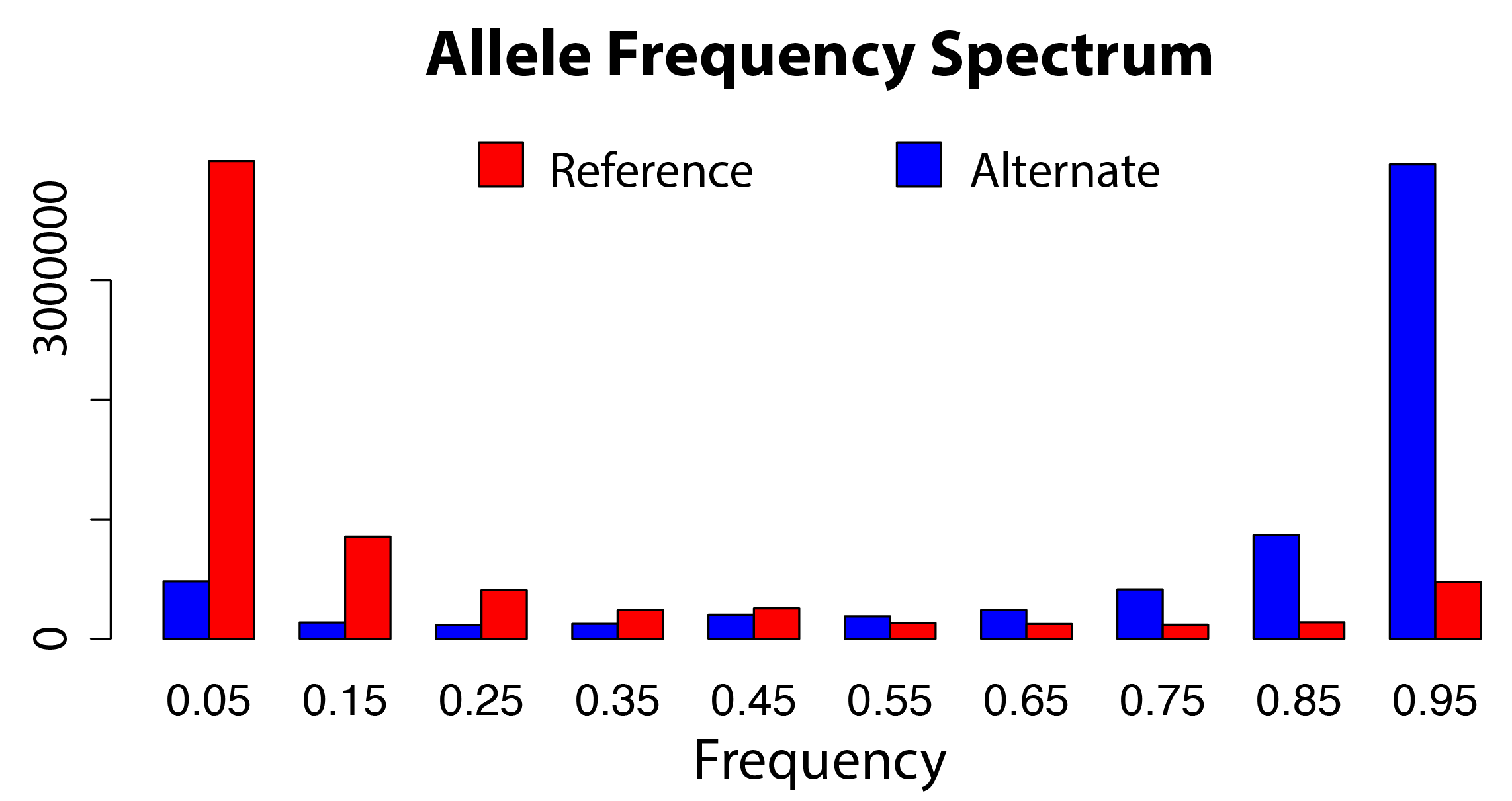


**Supplemental Figure 1:** The frequency spectrum of reference and alternative allele calls across identified polymorphic sites. Red represents calls matching the reference allele and blue represents calls alternative to the reference allele.


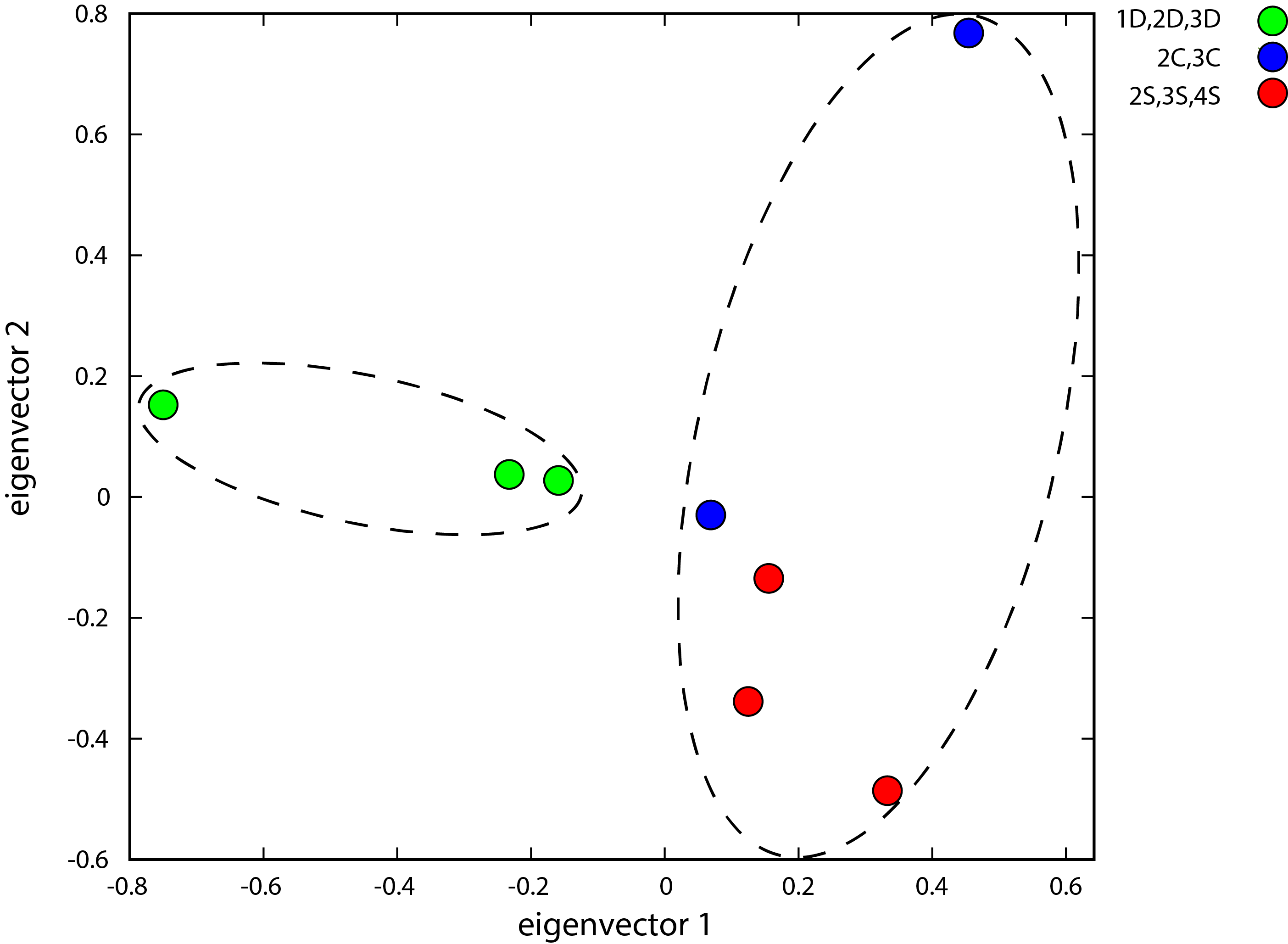


**Supplemental Figure 2:** Principal components analysis (PCA) plot showing that 1D, 2D and 3D (called "Desert") are separated by the first principal component (eigenvector) from 2S, 3S, 4S, 2C and 3C (called "Savannah"), suggesting that the two populations may be significantly distinct from one another.


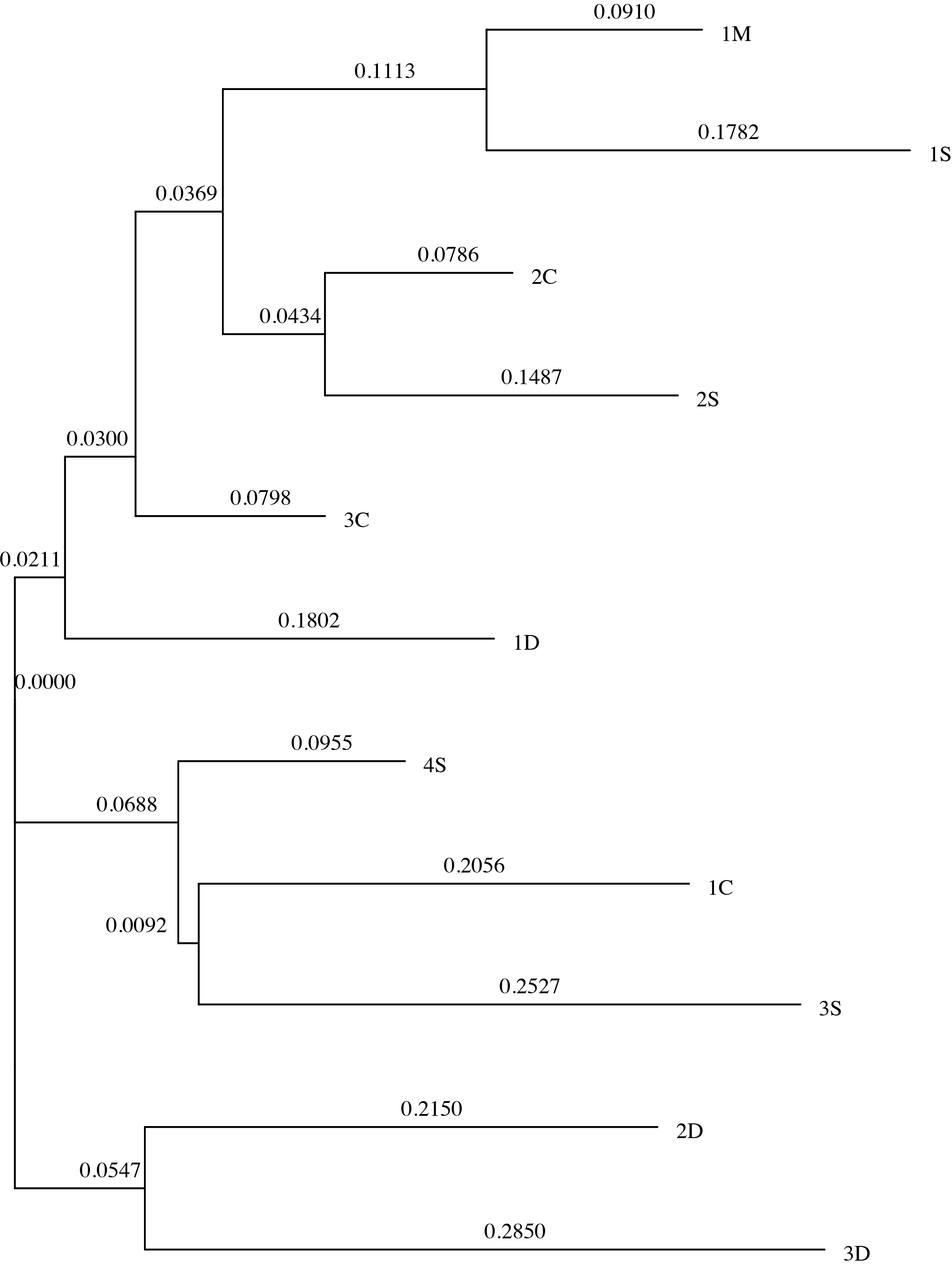


**Supplemental Figure 3:** Neighbor-joining phylogenetic tree constructed from polymorphisms located in the *Api m 6* region. Individuals do not form monophyletic clades according to the expected population structure.


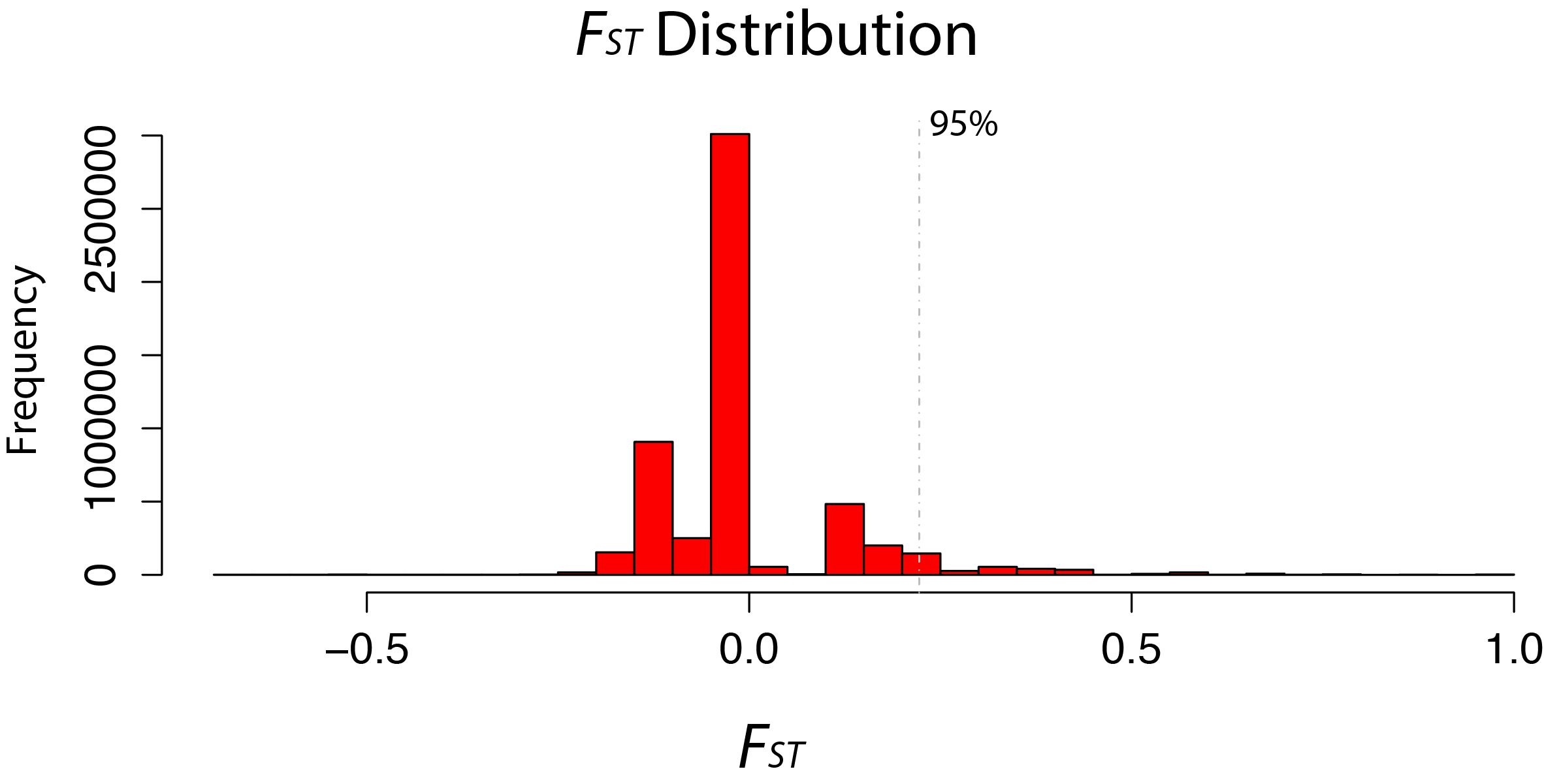


**Supplemental Figure 4:** Distribution of per-SNP-*F_ST_* values between Desert and Savannah populations. The vertical line represents the location of the 95^th^ percentile.


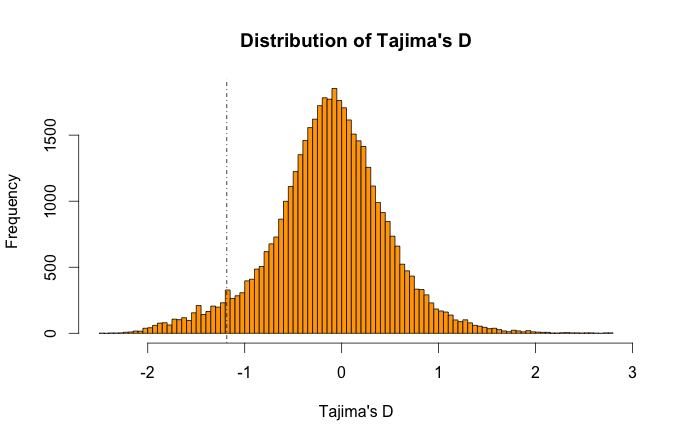


**Supplemental Figure 5:** Distribution of Tajima’s *D* values in 5kb non-overlapping windows. The vertical line represents the location of the 95^th^ percentile.

**References for the Supplement**

David Reich, Kumarasamy Thangaraj, Nick Patterson, Alkes L. Price, and Lalji Singh (2009) Reconstructing Indian population history. Nature 461:489-494, especially Supplement 2.

B. S. Weir and C. Clark Cockerham (1984) Estimating F-statistics for the analysis of population structure. Evolution 38:1358-1370.

Weir, B.S. (1996) Population substructure. Genetic data analysis II, pp. 161-173. Sinauer Associates, Sunderland, MA.

Eva-Maria Willing, Christine Dreyer, Cock van Oosterhout (2012) Estimates of genetic differentiation measured by FST do not necessarily require large sample sizes when using many SNP markers. PLoS One 7:e42649.

Sewall Wright (1951) The genetical structure of populations. Ann Eugen 15:323-354.
